# An integrative deep learning model based on dual-mode ultrasound for diagnosing gallbladder polyps

**DOI:** 10.1186/s13244-026-02213-8

**Published:** 2026-02-02

**Authors:** Congyu Tang, Yilei Shi, Lifan Wang, Xing Zhao, Chunlei Li, Peishan Guan, Zhidan Geng, Jianfei Chen, Qing Yu, Wenping Wang, Xiao Xiang Zhu, Haixia Yuan

**Affiliations:** 1 Department of Ultrasound, Putuo Hospital, Shanghai University of Traditional Chinese Medicine, Shanghai, China; 2 Department of Ultrasound, Zhongshan Hospital of Fudan University, Shanghai, China; 3 MedAI Technology (Wuxi) Co., Ltd., Wuxi, China; 4 Department of Ultrasound, Zhongshan Hospital(Xiamen), Fudan University, Shanghai, China; 5 Chair of Data Science in Earth Observation, Technical University of Munich, Munich, Germany

**Keywords:** Gallbladder, Gallbladder polyp, Ultrasound, Deep learning

## Abstract

**Objectives:**

The aim of this study was to develop an artificial intelligence model to automatically differentiate between non-neoplastic and neoplastic gallbladder polyps, while also distinguishing benign from malignant polyps.

**Materials and methods:**

Patients with gallbladder polyps who underwent cholecystectomy from January 2022 to June 2023 were recruited from two hospitals retrospectively. Conventional ultrasound findings and clinical characteristics of patients before cholecystectomy were acquired. Ultrasound image blocks of gallbladder lesions were automatically segmented by the Unet network for diagnosis. A fusion deep learning model based on dual-mode ultrasound (grey-scale ultrasound and colour Doppler flow imaging) was established to diagnose gallbladder polyps and validated in the validation and test set. Finally, we compared the diagnostic efficiency of the model with that of radiologists and guidelines.

**Results:**

A total of 339 patients (mean ages 53.17 ± 15.89, 182 females) were enroled in this study. The Dice coefficient and intersection over union (IoU) value of the automatic segmentation based on the Unet-efficientnet-b4 network were 0.912 and 0.838. In differentiating non-neoplastic from neoplastic polyps, the integrative deep learning (IDL) model showed area under the curves (AUCs) of 0.829 and 0.802 in validation and test sets, respectively. In differentiating benign and malignant polyps, the IDL model showed AUCs of 0.844 and 0.839 in validation and test sets, respectively. In the test set, the diagnostic performance of two junior radiologists was improved with the assistance of the IDL model.

**Conclusion:**

The IDL model based on dual-mode ultrasound could achieve accurate and automatic segmentation of gallbladder lesions, and showed excellent diagnostic performance for diagnosing gallbladder polyps.

**Critical relevant statement:**

We developed a deep learning model based on conventional ultrasound that performs gallbladder segmentation while differentiating neoplastic from non-neoplastic polyps and benign from malignant polyps.

**Key Points:**

- Diagnosing gallbladder polyps through a deep learning model based on conventional ultrasound presents challenges.
- IDL model enables automated segmentation of the gallbladder and diagnosis of gallbladder polyps.
- The IDL model is a reliable tool to assist junior radiologists in diagnosis and has potential for reducing unnecessary cholecystectomies.

**Graphical Abstract:**

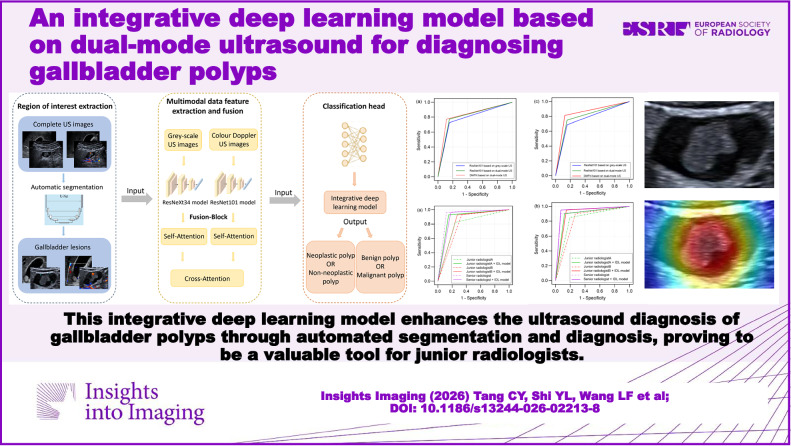

## Introduction

With the development of ultrasound and the popularity of health examination, the detection rate of gallbladder polyps in the population is approximately 5–10% [1–3]. Gallbladder polyps generally refer to mucosal lesions that protrude into the gallbladder lumen, and can be classified into non-neoplastic polyps (such as cholesterol polyps and inflammatory polyps) and neoplastic polyps (such as adenomas and GB carcinomas) [4]. Gallbladder adenomas have a malignant tendency, potentially progressing to gallbladder carcinoma, which is a highly malignant tumour with a five-year survival rate of only 5–10% [5, 6]. Therefore, the identification based on images of neoplastic polyps and early gallbladder carcinoma is crucial for patient management and prognosis.

Conventional ultrasound, including grey-scale ultrasound and colour Doppler flow imaging (CDFI), is the most common method to diagnose gallbladder disease and is accessible across all levels of hospitals. However, diagnosing gallbladder polyps only through conventional ultrasound requires certain requirements for the experience of ultrasound radiologists. In clinical practice and guidelines, size ≥ 10 mm is generally used as the surgical indicator for gallbladder polyps [7]. However, this will result in a large number of unnecessary cholecystectomies, and it may also lead to the missed diagnosis of small neoplastic polyps. The Society of Radiologists in Ultrasound (SRU) proposed ultrasound guidelines for gallbladder polyps in 2022, further improving the risk stratification [8]. We also established a risk stratification model based on conventional ultrasound for gallbladder polyps in a previous study [9]. However, current models still cannot achieve automated identification and diagnosis of gallbladder polypoid lesions.

Deep learning and radiomics have shown excellent diagnostic performance in medical imaging, and have applications in the field of gallbladder diseases [10–15]. Therefore, it is necessary to develop a deep learning model based on conventional ultrasound to identify gallbladder polyps automatically. In our previous study [16], we established a machine learning‑based ultrasound radiomics model to identify gallbladder mass and achieved excellent results. On this basis, this study intends to add the CDFI mode to build a dual-mode model and focus on the difficult field of gallbladder mass to accurately diagnose gallbladder polypoid lesions.

In our study, we developed an integrative deep learning (IDL) model to automatically segment gallbladder polyps in ultrasound images and provide preoperative predictions, and we aim to achieve accurate differentiating non-neoplastic polyps from neoplastic polyps and benign from malignant polyps differentiation, and reduce the unnecessary cholecystectomy rate.

## Materials and methods

This retrospective study was approved by the ethics committees of Zhongshan Hospital of Fudan University (No: B2022-187R) and Putuo Hospital, Shanghai University of Traditional Chinese Medicine (PTEC-A-2025-33(S)-1). Written informed consent was exempted due to the retrospective nature of the study.

### Patients

From January 2022 to June 2023, a total of 334 consecutive patients were enrolled from Institution1 (Zhongshan Hospital of Fudan University) and Institution2 (Zhongshan Hospital of Fudan University Xiamen Branch). The inclusion criteria were as follows: (a) patients who underwent cholecystectomies and pathologically confirmed gallbladder polyps; (b) complete clinical baseline data and intact ultrasound images. A total of 56 patients were excluded based on the following criteria: (a) incomplete clinical data (*n* = 14); (b) lack or poor quality of ultrasound images (*n* = 35); (c) other malignant diseases of the digestive system (*n* = 7). In total, 278 patients were included in our study, and 85% of the patients were randomly chosen as the training set, with the remaining 15% used as the validation set.

**Fig. 1:**
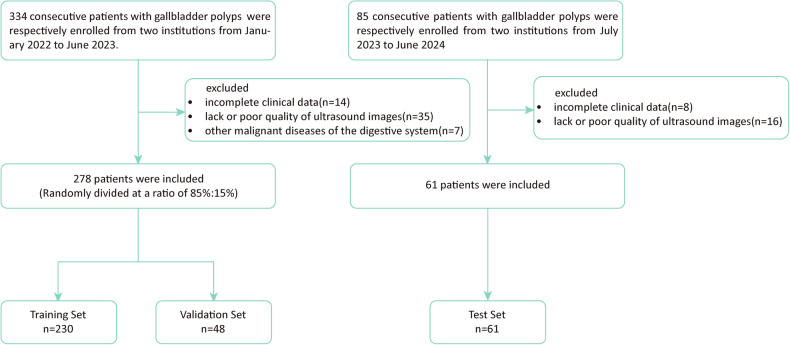
Flowchart of the enrolment process

Following the same inclusion and exclusion criteria, we set up an independent test set for model validation. From July 2023 to December 2024, 61 consecutive patients with gallbladder polyps were enroled from Institution1 (Zhongshan Hospital of Fudan University) and Institution3 (Putuo Hospital, Shanghai University of Traditional Chinese Medicine) as the test cohort. A detailed flowchart of the patient selection process is displayed in Fig. 1

Clinical data were collected from medical records, including pathological type of gallbladder polyps, age, sex, gastrointestinal symptoms, preoperative carcinoembryonic antigen (CEA) and carbohydrate antigen 199 (CA199) value.

### Ultrasound examination and characteristics

The details of ultrasound examination and equipment are provided in Appendix 1. Grey-scale ultrasound and CDFI were performed for all patients before surgery. A radiologist (C.T.) with 3 years of abdominal US experience independently assessed the US characteristics and was blinded to the pathological results and clinical characteristics. The following US characteristics were assessed: (1) number of polyps(single/multiple); (2) colour Doppler signal (present/absent); (3) echogenicity (hypoechoic/isoechoic/hyperechoic); (4) gallbladder stone (present/absent); (5) stalk of polyps (pedunculated with thin stalk or ball on the wall/ pedunculated with wide or thick stalk or sessile); (6) focal wall thickening ≥ 4 mm adjacent to polyps (present/absent). Stalk of polyps and focal gallbladder wall thickening were defined based on the Society of Radiologists in Ultrasound consensus conference of gallbladder polyp guideline [8].

### Data preprocessing

In our study, we conducted detailed data preprocessing on grey-scale ultrasound and colour Doppler ultrasound images to enhance model performance and classification accuracy. The detailed preprocessing operations and experimental code are provided in Appendix 2.

The US images of the training cohort and validation cohort were manually segmented by a US radiologist (C.T.) with 3 years of abdominal US experience, and the regions of interest (ROI) of lesions were defined as the outer edge of the gallbladder wall. US and clinical characteristics were included in the univariate analysis and multivariate logistic regression analyses through forward stepwise selection.

### Image segmentation and deep learning models

**Fig. 2:**
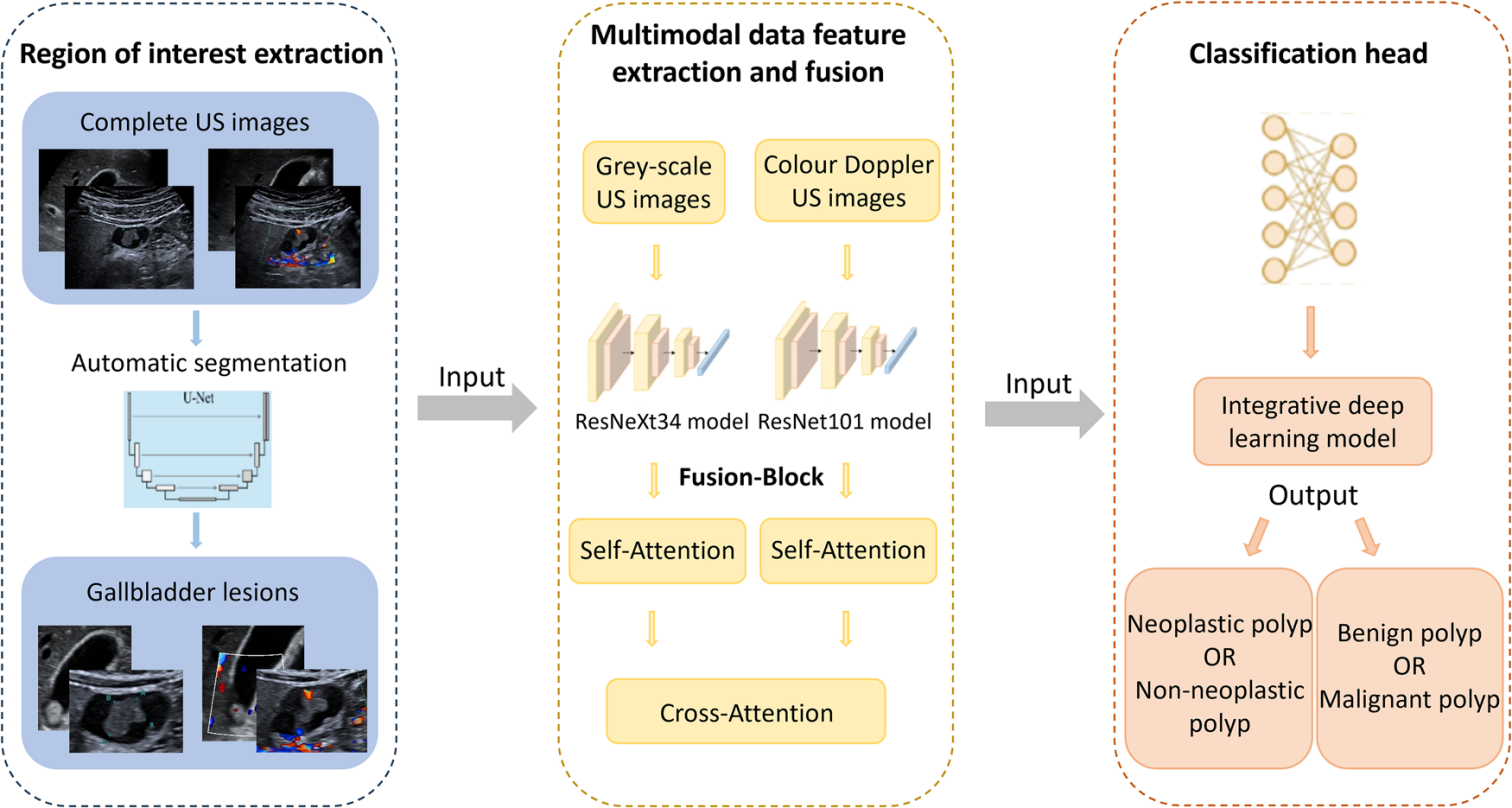
Workflow of the IDL model

We established a classification network architecture based on dual-mode ultrasound, as illustrated in Fig. 2. The architecture comprises three core components: ROI extraction, multimodal data feature extraction and fusion, and the classification head.

#### Image segmentation

We utilised a U-net network to process the input medical images, obtaining segmentation results and extracting ROIs from both grey-scale and colour Doppler images. The process of extracting ROIs is described in Appendix 3.

#### Deep multimodal fusion network (DMFN)

To integrate grey-scale ultrasound and CDFI, we designed a DMFN. Initially, feature vectors were extracted from the two different modalities (grey-scale ultrasound and CDFI) using two independent deep learning models. Specifically, features from grey-scaleultrasound images were extracted using a ResNeXt34 model pre-trained on ImageNet, while features from colour Doppler images were extracted using a ResNet101 model pre-trained on ImageNet. These extracted features were then fed into the fusion module (denoted as “Fusion-Block” in Fig. 2), where feature fusion is performed using a sophisticated attention mechanism. Specifically, we first apply self-attention to each of the two feature streams independently, allowing for intra-modal feature refinement. Subsequently, we employ cross-attention between the two streams, enabling effective inter-modal information exchange. This hierarchical attention approach facilitates comprehensive feature interaction and integration. The detailed process of this multi-stage feature fusion is elaborated in Appendix 4. The resulting fused features, which encapsulate rich information from dual-mode ultrasound, were then input into the classification head to achieve classification of neoplastic/non-neoplastic and benign/malignant gallbladder polyps.

#### Model interpretability

To investigate the interpretability of our IDL model, we applied a Gradient-weighted class activation mapping (Grad-CAM) to convert the feature maps into colour images. This approach efficiently localises critical regions of model interest and visualises the decision rationale of the classification network, thereby enhancing interpretability analysis for classification tasks. Implementation details are provided in the Appendix 5. In the heatmap with pseudo-colour mapping, warmer colours (e.g., red, yellow) indicate regions with higher contributory relevance to target classification, while cooler colours (e.g., blue) denote areas of lower relevance or non-contribution.

### US image interpretation and computer-aided diagnosis

To evaluate human readers' diagnostic performance for neoplastic polyps, three US radiologists independently read the original US images of patients in the test cohort and determined a differential diagnosis (non-neoplastic or neoplastic polyp). Two junior radiologists (Z.D.G., J.F.C.) with 3 years and 1 year of abdominal US and a senior radiologist (H.X.Y.) with more than 15 years of abdominal US were blinded to the pathological results. Second, radiologists determine differential diagnosis with results generated by the IDL model again 2 weeks later, and US images of patients are presented in a randomised order to decrease recall bias. To assess the diagnostic performance of radiologists in differentiating benign and malignant polyps, the identical approach described above was repeated for evaluation.

### Evaluation of the Society of Radiologists in Ultrasound (SRU) gallbladder polyp consensus conference guideline

Characteristics of criteria in the SRU guideline were collected, and polyps were classified according to the guidelines. We judged the polyps that suggested surgical consultation in the guidelines as requiring surgical intervention, and the remaining polyps as not requiring it (requiring surgical intervention: polyps ≥15 mm in the Extremely low risk group, polyps ≥ 10 mm in the low risk group, polyps with focal wall thickening ≥6 mm in the indeterminate risk group). And according to the guidelines, the cases identified as neoplastic polyps in the IDL model were considered to require surgical intervention and compared with the SRU guidelines.

### Model implementation and statistical analysis

In the ROI extraction experiments, we optimised the model during training using a composite loss function that combines Dice loss and cross-entropy loss. The Adam optimiser was employed with a learning rate set to 0.0001, and a learning rate decay strategy was implemented, halving the learning rate every 50 epochs if the validation loss did not improve. The batch size was set to 32, and training was conducted for 200 epochs, with early stopping applied if the validation loss did not improve for 20 consecutive epochs. The primary evaluation metrics were the Dice coefficient (Dice) and intersection over union (IoU).

In the multimodal fusion classification experiments, we optimised the classifier using the cross-entropy loss function and the Adam optimiser, with a learning rate set to 0.001. The batch size was set to 32, and training was conducted for 200 epochs. All experiments were conducted using Python and the PyTorch framework, with training and evaluation performed on a system equipped with an NVIDIA GTX 4090 Ti GPU to accelerate computations.

SPSS 25.0 software (IBM Corporation, Armonk) and R version 4.5.0 were used for statistical analysis in this study. In the univariate analysis, Student’s t-test or Mann-Whitney U test was performed for continuous variables. Chi-square test or Fisher’s exact test was used for categorical variables. Forward stepwise logistic regression analysis was used for the multivariate analysis. The diagnostic performance of the model was described by the AUC. The DeLong’s test was used to assess the differences between the AUCs. *p* < 0.05 was considered statistically significant.

## Results

In total, 339 patients were enroled in our study. There were 230, 48 and 61 patients in the training set, validation set, and test set, respectively, including 785 grey-scale ultrasound images and 524 colour Doppler ultrasound images. The characteristics of patients in each set are summarised in Table 1.

**Table 1:** The baseline of patients with gallbladder polyps in this study

Characteristics	Training cohort (*n* = 230)	Validation cohort (*n* = 48)	Test cohort (*n* = 61)
Clinical characteristics			
Pathological type			
Non-neoplastic polyps	117 (50.9)	26 (54.2)	33 (54.1)
Adenoma	46 (20.0)	6 (12.5)	8 (13.1)
Adenoma with carcinoma	23 (10.0)	7 (14.6)	11 (18.0)
Gallbladder cancer	44 (19.1)	9 (18.8)	9 (14.8)
Sex			
Female	131 (57.0)	29 (60.4)	22 (36.1)
Male	99 (43.0)	19 (39.6)	39 (63.9)
Age (y)	53.63 ± 15.86	52.25 ± 16.40	52.64 ± 16.01
CA199 (U/mL)	9.60 (6.08, 16.38)	8.25 (4.93, 15.50)	8.00 (4.63, 12.43)
CEA (ng/mL)	1.80 (1.20, 2.60)	1.85 (1.00, 2.88)	1.40 (1.08, 2.20)
Gastrointestinal symptom			
Present	81 (35.2)	20 (41.7)	16 (26.2)
Absent	149 (64.8)	28 (58.3)	45 (73.8)
US characteristics			
Size of polyps (mm)	15.66 ± 8.80	16.15 ± 9.72	16.03 ± 7.61
Number			
Single	144 (62.6)	33 (68.8)	38 (62.3)
Multiple	86 (37.4)	15 (31.3)	23 (37.7)
Colour Doppler signal			
Present	142 (61.7)	30 (62.5)	34 (55.7)
Absent	88 (38.3)	18 (37.5)	27 (44.3)
Echogenicity			
Hypoechoic	73 (31.7)	17 (35.4)	15 (24.6)
Isoechoic	112 (48.7)	29 (60.4)	35 (57.4)
Hyperechoic	45 (16.9)	2 (4.2)	11 (18.0)
Gallbladder stone			
Present	55 (23.9)	12 (25.0)	10 (16.4)
Absent	175 (76.1)	36 (75.0)	51 (83.6)
Stalk of polyps			
Thin stalk or ball on the wall	96 (41.7)	27 (56.3)	23 (37.7)
Wide/thick stalk or sessile	134 (58.3)	21 (43.7)	38 (62.3)
Focal wall thickening			
Present	46 (20.0)	9 (18.8)	11 (18.0)
Absent	184 (80.0)	39 (81.3)	50 (82.0)

*CA199* carbohydrate antigen 199, *CEA* carcinoembryonic antigen

In the multivariable logistic regression analysis, age (OR = 1.03, 95% CI: 1.00–1.06, *p* = 0.02), size (OR = 1.31, 95% CI: 1.18–1.45, *p* < 0.01), gallbladder stone (OR = 3.50, 95% CI: 1.16–10.59, *p* = 0.03), wide/thick stalk or sessile (OR = 4.94, 95% CI: 2.15–11.38, *p* < 0.01) were significant predictors for neoplastic polyps (Table S1). For malignant polyps, age (OR = 1.03, 95% CI: 1.00–1.06, *p* = 0.045), size (OR = 1.20, 95% CI: 1.11–1.30, *p* < 0.01), wide/thick stalk or sessile (OR = 5.64, 95% CI: 1.17–27.26, *p* = 0.03), focal wall thickening (OR = 4.38, 95% CI: 1.42–13.52, *p* = 0.01) (Table S2).

### Segmentation model performance

Detailed performance for different segmentation models in the validation set is presented in Table 2. The Dice and IoU of Unet-efficientnet-b4 were 0.912 and 0.838, respectively, showing superior efficiency compared to other network models, which were used for the segmentation of all gallbladder ultrasound images in our study.

**Table 2:** Performance of the segmentation model in the validation set

Segmentation model	Dice coefficient	IoU
Unet- efficientnet-b4	0.912	0.838
Unet-resnet34	0.892	0.814
Unet-resnet50	0.903	0.825

*IoU* intersection over union

### Construction and validation of a deep learning model

**Fig. 3:**
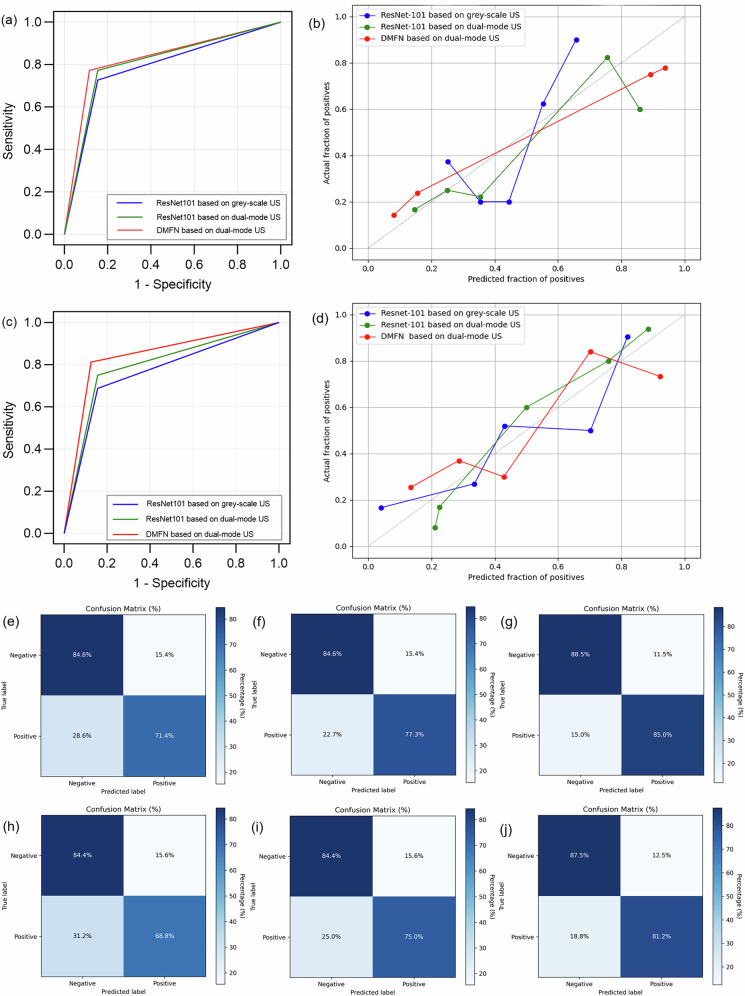
The diagnostic performance of each deep learning model in the validation set. **a**, **b** The receiver operating characteristic curves and calibration curves of the three models for neoplastic polyps in the validation set. **c**, **d** The receiver operating characteristic curves and calibration curves of the three models for malignant polyps in the validation set. **e**–**g** The confusion matrix of the three models for neoplastic polyps in the validation set (ResNet101 based on grey-scale US, ResNet101 based on dual-mode US and DMFN based on dual-mode US, respectively). **h**–**j** The confusion matrix of the three models for malignant polyps in the validation set (ResNet101 based on grey-scale US, ResNet101 based on dual-mode US and DMFN based on dual-mode US, respectively)

In differentiating non-neoplastic from neoplastic polyps, the AUC of the ResNet101 network was 0.787 in grey-scale ultrasound, and increased to 0.809 in dual-mode ultrasound. The DMFN (ResNet101+ ResNeXt34) based on dual-mode ultrasound showed excellent diagnostic efficiency of neoplastic polyps (AUC = 0.829), which was superior to other networks we established (Table 3). And the Brier score of the DMFN model was 0.155. The ROC curves and the calibration curves of the three models for the validation set are exhibited in Fig. 3a, b.

**Table 3:** The diagnostic performance for neoplastic polyps of each model in the validation set

Deep learning model	AUC	Sensitivity	Specificity	Accuracy of patients	Accuracy of images
Model based on grey-scale US Resnet101	0.787 (0.650–0.924)	0.727 (16/22)	0.846 (22/26)	0.792 (38/48)	0.790 (83/105)
Model based on dual-mode US ResNet101	0.809 (0.679–0.940)	0.773 (17/22)	0.846 (22/26)	0.813 (39/48)	0.810 (85/105)
Fusion model based on dual-mode US DMFN (resnet101 + resnet34)	0.829 (0.703–0.955)	0.773 (17/22)	0.885 (23/26)	0.836 (40/48)	0.827 (87/105)

*AUC* area under curve, *DMFN* deep multimodal fusion network

In the test set, the IDL model, composed of a segmentation model and DMFN network, had good performance in the diagnosis of neoplastic polyps (AUC = 0.802).

In differentiating benign from malignant polyps, the ResNet101 network achieved an AUC of 0.766 in grey-scale ultrasound, while demonstrating an increased diagnostic performance (AUC = 0.797) when applied to dual-mode ultrasound. Notably, the DMFN model exhibited superior diagnostic efficacy for malignant polyps in dual-mode ultrasound evaluations as well, attaining a significantly higher AUC value of 0.844 (Table 4) and a Brier score of 0.148. The ROC curves and the calibration curves of the three models for the validation set are exhibited in Fig. 3c, d. In the test set, our DMFN model showed good performance in the diagnosis of malignant polyps as well, with an AUC of 0.839.

**Table 4:** The diagnostic performance for malignant polyps of each model in the validation set

Deep learning model	AUC	Sensitivity	Specificity	Accuracy of patients	Accuracy of images
Model based on grey-scale US Resnet101	0.766 (0.612–0.919)	0.688 (11/16)	0.844 (27/32)	0.792 (38/48)	0.829 (87/105)
Model based on dual-mode US ResNet101	0.797 (0.652–0.942)	0.750 (12/16)	0.844 (27/32)	0.813 (39/48)	0.819 (86/105)
Fusion model based on dual-mode US DMFN (resnet101 + resnet34)	0.844 (0.713–0.974)	0.813 (13/16)	0.875 (28/32)	0.854 (41/48)	0.876 (92/105)

*AUC* area under curve, *DMFN* deep multimodal fusion network

### Comparison between the IDL models and a radiologist with different experience

**Fig. 4:**
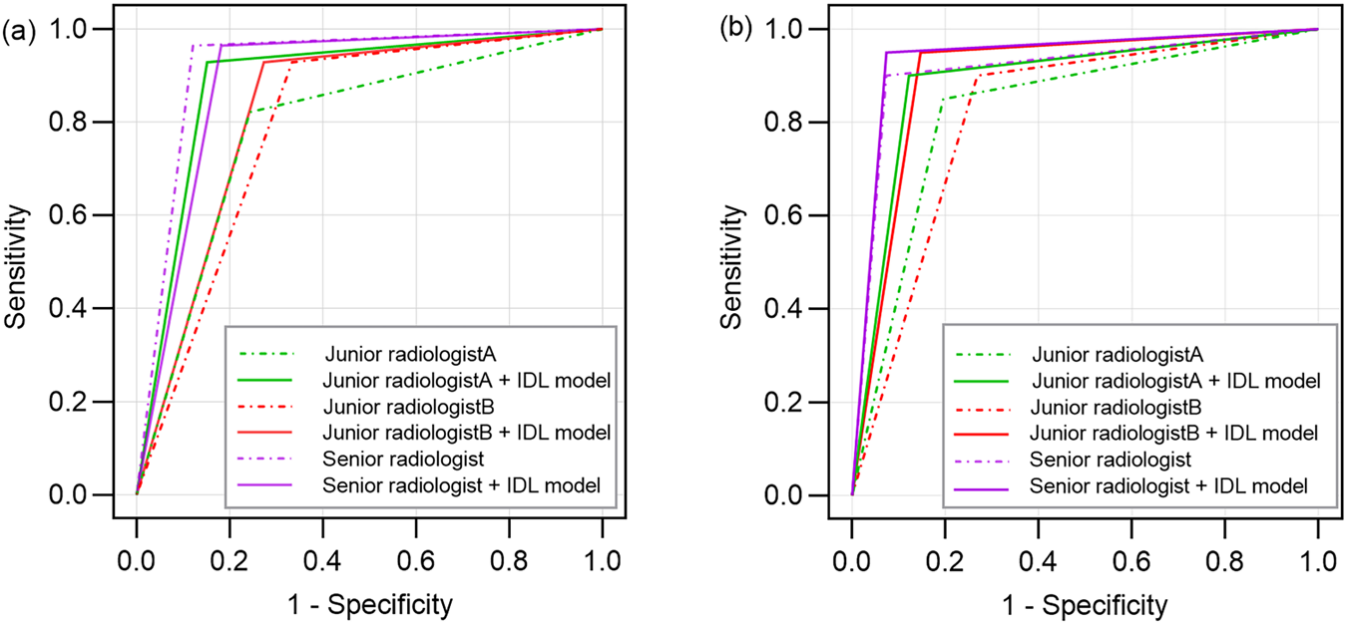
Diagnostic performance of radiologists with versus without IDL model assistance. **a** The receiver operating characteristic curves for differentiating neo-neoplastic and neoplastic polyps in the test set. **b** The receiver operating characteristic curves for differentiating benign and malignant polyps in the test set

In differentiating non-neoplastic from neoplastic polyps, the diagnostic performance of the IDL model was slightly superior to that of the two junior radiologists, but lower than that of the senior radiologist in the test set (*p* > 0.05) (Table 5). With the assistance of IDL model, the diagnostic performance of both junior radiologists improved, and the improvement of junior radiologist B was statistically significant (AUC_A_: 0.798 vs 0.828, *p* > 0.05 and AUC_B_: 0.790 vs 0.889, *p* = 0.03) (Fig. 4a). Similarly, in distinguishing benign from malignant polyps, the IDL model demonstrated diagnostic performance intermediate between that of the two junior radiologists and the senior radiologist in the test set (*p* > 0.05) (Table 5). Following assistance from the IDL model, the diagnostic efficiency of both junior radiologists improved. A statistically significant improvement was observed for junior radiologist B (AUC_A_: 0.827 vs 0.889, *p* > 0.05 and AUC_B_: 0.816 vs 0.914, *p* = 0.03) (Fig. 4b).

**Table 5:** The diagnostic performance of radiologists in differentiating gallbladder polyps

	AUC	Accuracy	Sensitivity	Specificity	PPV	NPV	*p* value
Non-neoplastic and neoplastic polyps							
IDL model	0.802 (0.700–0.904)	80.3% (49/61)	78.6% (22/28)	81.8% (27/33)	78.6% (22/28)	81.8% (27/33)	
Radiologists							
Junior radiologist A	0.798 (0.703–0.893)	78.7% (48/61)	92.9% (26/28)	66.7% (22/33)	70.3% (26/37)	91.7% (22/24)	0.94^a^
Junior radiologist B	0.790 (0.686–0.893)	78.7% (48/61)	82.1% (23/28)	75.8% (25/33)	74.2% (23/31)	83.3% (25/30)	0.85^a^
Senior radiologist	0.922 (0.855–0.988)	91.8% (56/61)	96.4% (27/28)	87.9% (27/33)	87.1% (27/31)	96.7% (29/30)	0.06^a^
Radiologists with IDL-aid							
Junior radiologist A + IDL	0.828 (0.737–0.919)	82.0% (50/61)	92.9% (26/28)	72.7% (24/33)	74.3% (26/35)	92.3% (24/26)	0.48^b^
Junior radiologist B + IDL	0.889 (0.810–0.967)	88.5% (54/61)	92.9% (26/28)	84.8% (28/33)	83.9% (26/31)	93.3% (28/30)	0.03^b^
Senior radiologist + IDL	0.891 (0.816–0.967)	88.5% (54/61)	96.4% (27/28)	81.8% (27/33)	81.8% (27/33)	96.4% (27/28)	0.32^b^
Benign and malignant polyps							
IDL model	0.839 (0.736–0.942)	85.2% (52/61)	80.0% (16/20)	87.8% (36/41)	76.2% (16/21)	90.0% (36/40)	
Radiologists							
Junior radiologist A	0.827 (0.726–0.929)	82.0% (50/61)	85.0% (17/20)	80.5% (33/41)	68.0% (17/25)	91.7% (33/36)	0.86^a^
Junior radiologist B	0.816 (0.720–0.912)	78.7% (48/61)	90.0% (18/20)	73.2% (30/41)	62.1% (18/29)	93.8% (30/32)	0.71^a^
Senior radiologist	0.913 (0.835–0.992)	91.8% (56/61)	90.0% (18/20)	92.7% (38/41)	85.7% (18/21)	95.0% (38/40)	0.28^a^
Radiologists with IDL-aid							
Junior radiologist A + IDL	0.889 (0.805–0.973)	88.5% (54/61)	90.0% (18/20)	87.8% (36/41)	78.3% (18/23)	94.7% (36/38)	0.09^b^
Junior radiologist B + IDL	0.914 (0.844–0.985)	90.2% (55/61)	95.0% (19/20)	87.8% (36/41)	79.2% (19/24)	97.3% (36/37)	0.03^b^
Senior radiologist + IDL	0.938 (0.875–1.000)	93.4% (57/61)	95.0% (19/20)	92.7% (38/41)	86.4% (19/22)	97.4% (38/39)	0.32^b^

*AUC* area under curve, *PPV* positive predictive value, *NPV* negative predictive value, *IDL* integrative deep learning

^a^ *p* value of AUCs differ between the radiologists and the IDL model

^b^ *p* value of AUCs differs between the radiologists before and after IDL model assistance

**Fig. 5:**
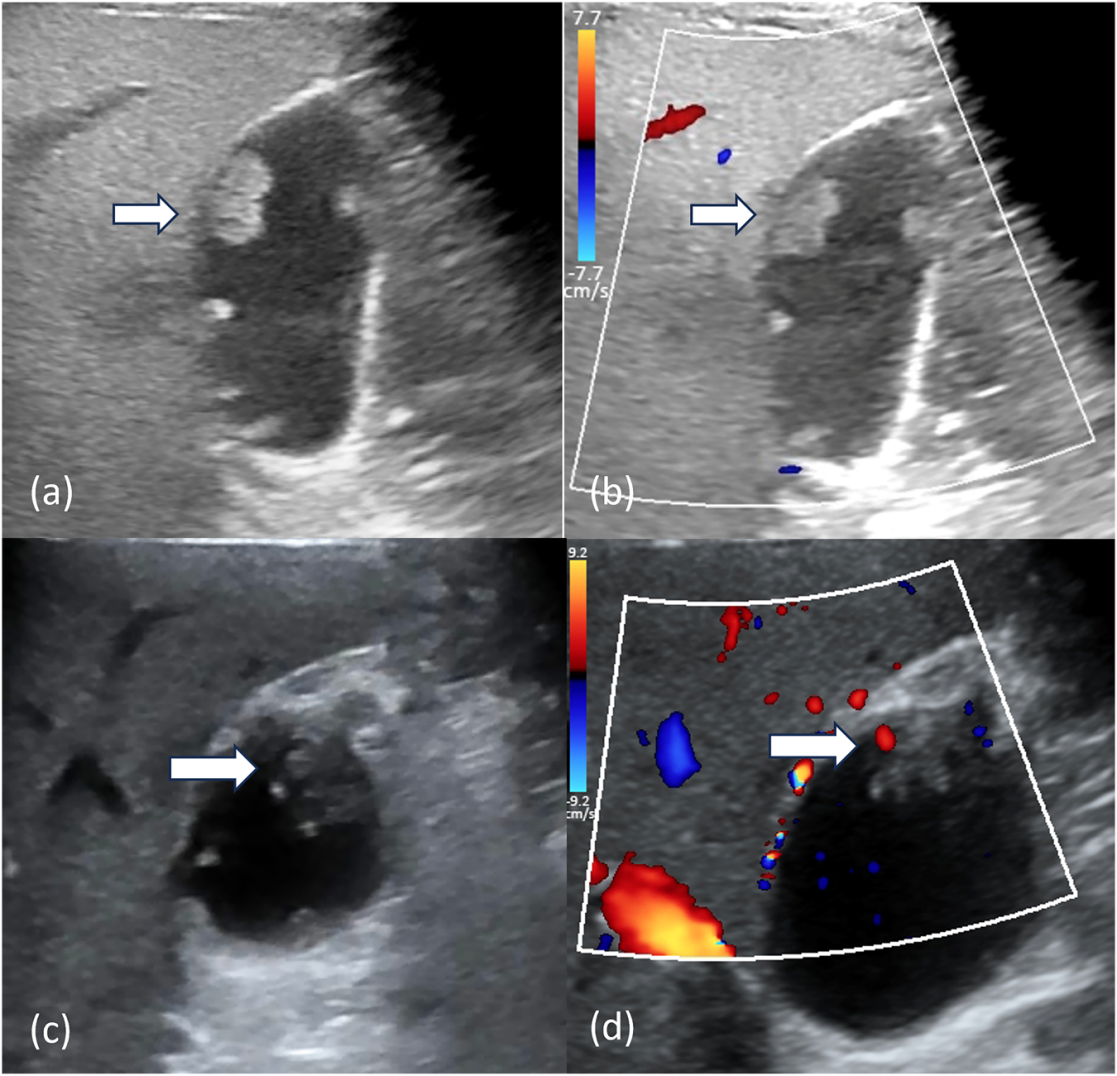
Examples correctly diagnosed by the IDL model. **a**, **b** A case of a 37-year-old male with gallbladder adenoma. The largest polyp (arrow) was correctly diagnosed by the IDL model, but was misdiagnosed by a junior radiologist. **c**, **d** A case of a 70-year-old female with cholesterol polyps. The polyp (arrow) was correctly diagnosed by the IDL model, but was misdiagnosed by a junior radiologist and the senior radiologist

For inter-observer agreement between the two junior radiologists, κ-values were 0.605 (95% CI: 0.409–0.801) in differentiating non-neoplastic from neoplastic polyps and 0.802 (95% CI: 0.653–0.951) in distinguishing benign from malignant polyps. Following implementation of the IDL model, these κ-values improved to 0.803 (95% CI: 0.654–0.952) and 0.863 (95% CI: 0.734–0.992), respectively. Representative cases demonstrating diagnostic discordance between IDL predictions and radiologists are presented in Fig. 5.

### Interpretation of the model

**Fig. 6:**
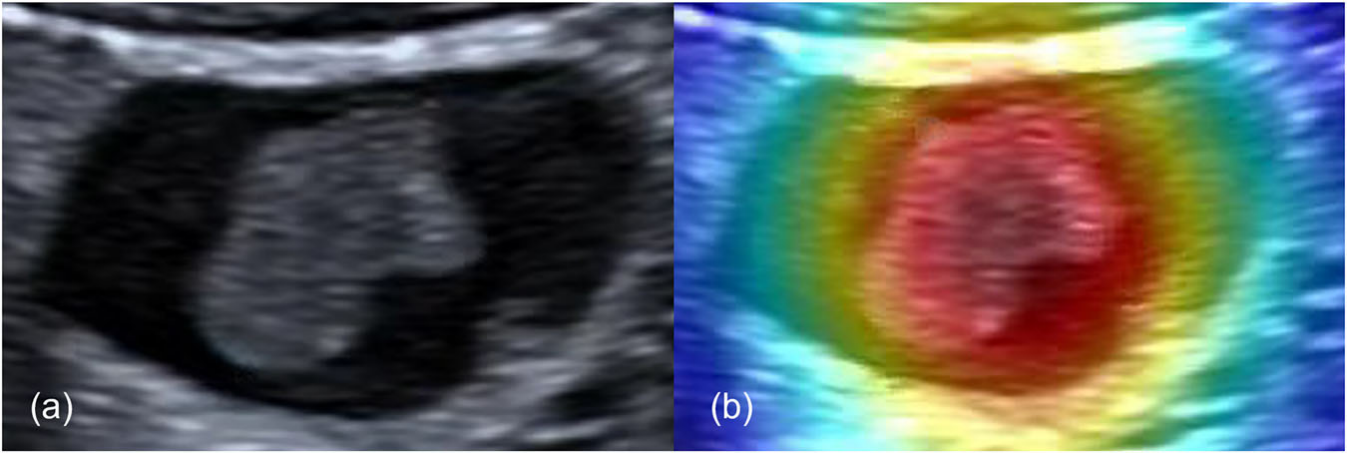
Representative images of gallbladder polyps in Grad-CAM. **a** A case of gallbladder adenomas with high-grade intraepithelial neoplasia (carcinoma in situ). **b** Pseudo-colour image with Grad-CAM

Figure 6 demonstrates the application of Grad-CAM. The IDL model could identify gallbladder polyps and mainly paid attention to the information inside the polyp.

### Clinical utility of IDL model: potential for reducing unnecessary cholecystectomy

In the validation set and the test set, the unnecessary cholecystectomy rates (proportion of polyps predicted to be neoplastic that were actually non-neoplastic) of the IDL model was 15.0% and 21.4%, respectively, which were lower than SRU guidelines and Size (Table S3). The pre-specified operating points were set as 0.5, and the calibration curve of the IDL model in the test set are presented in Fig. S1a, with a Brier score of 0.140. The decision curve analysis illustrated the net benefit of clinical utility in the three models in the test set (Fig. S1b).

## Discussion

This study demonstrates that the fusion model based on dual-mode ultrasound achieves accurate segmentation of gallbladder lesions and exhibits excellent diagnostic performance in differentiating neoplastic from non-neoplastic polyps (AUC = 0.829) as well as benign from malignant polyps (AUC = 0.844). Furthermore, the IDL model can enhance the diagnostic capabilities of junior radiologists.

Most of the studies on deep learning for gallbladder polyps only included grey-scale ultrasound images [17–19]. We added colour Doppler ultrasound images to provide the model with blood flow information around and inside the lesion, which further improved the diagnostic efficiency of the model and did not reduce the applicability. Critically, the IDL model provides diagnostic support to junior radiologists for identifying neoplastic and malignant polyps, demonstrating particular value in resource-limited areas where advanced imaging modalities (e.g., contrast-enhanced ultrasound or MRI) are unavailable.

Our IDL model not only captures and integrates multimodal information comprehensively, significantly improving classification accuracy, but also enhances the robustness and optimisation of feature representation. Our model begins by extracting the ROI from the images, which improves classification accuracy by eliminating irrelevant background information, allowing the classification model to focus on the target regions, enhancing accuracy and robustness and reducing computational cost. And it enhances model interpretability, as analysing specific regions enables a clear explanation of the model’s predictions. Meanwhile, Grad-CAM is applied to our images to further improve the interpretability of the model [20]. In our multimodal fusion network, we designed a fusion block with the following advantages: Firstly, the self-attention mechanism captures long-range dependencies within each modality, allowing the model to better understand both local and global information within each modality. This effectively suppresses noise within each modality, and improves the robustness of feature representation. Secondly, the cross-attention mechanism captures correlations between different modalities, enabling the model to combine complementary information from grey-scaleand blood flow images.

Three radiologists with different experiences were included in our study, the diagnostic efficiency of our IDL model for neoplastic polyps and malignant polyps was slightly higher than that of the two junior radiologists, but the difference was not statistically significant (*p* > 0.05). It could be considered that the IDL model at least reached the diagnostic level of junior radiologists. With the aid of the IDL model, the diagnostic efficiency of two junior radiologists was improved to varying degrees, and one of them was statistically significant (*p* < 0.05), suggesting that our IDL model is valuable for assisting junior radiologists in diagnosing gallbladder neoplastic polyps and malignant polyps, and can improve the diagnostic performance of junior radiologists or physicians in remote areas with poor medical resources.

In our study, age, size of polyps, gallbladder stone, shape of stalk, and focal gallbladder wall thickening are the risk factors of neoplastic polyps or malignant polyps, which were consistent with previous studies [21, 22]. Focal gallbladder wall thickening is recognised as a significant risk-stratification feature in the SRU guidelines [8]. Our study further confirms its status as an independent risk factor for malignant polyps, aligning with previous literature [23, 24]. At present, the size of the polyp is still an important criterion for cholecystectomy, and the current clinical guidelines recommend 10 mm as the cut-off value [1, 7, 25]. In the SRU guidelines, the size of the polyp, shape of the stalk and focal wall thickening were used as criteria [8]. However, in our study, the proportions of non-neoplastic polyps among those recommended for surgical consultation based on the two aforementioned criteria (i.e., polyp size alone and the SRU guidelines) in the test set reached 40.9% and 45.1%, respectively, significantly higher than those of our IDL model (21.4%). It is suggested that our model holds potential benefits for avoiding overtreatment when compared with current clinical guidelines; however, its efficacy still requires further validation in a prospective validation cohort.

The study had several limitations. Firstly, selection biases cannot be avoided because of the retrospective nature of the study. Furthermore, only patients who underwent cholecystectomy were included, while a substantial number of patients with polyps who did not undergo surgery were excluded, which may introduce additional selection bias in the enroled data; we will conduct long-term prospective follow-up in subsequent studies to further validate the model’s diagnostic efficacy. Additionally, the radiologist who annotated the dataset was also the one who assessed the US characteristics, which may introduce further selection bias. Second, as a study on deep learning models, the sample size was small, and larger multi-centre and prospective studies are needed in the future. In addition, our study only used ultrasound images rather than ultrasound clips, which may lead to information loss.

In conclusion, the IDL model we established could identify and segment gallbladder lesions automatically, and had excellent diagnostic performance for neoplastic polyps and malignant polyps, which hold potential benefits for avoiding overtreatment.

## Supplementary information

ELECTRONIC SUPPLEMENTARY MATERIAL

## Data Availability

The patient-derived medical imaging data used in this study contain sensitive personal health information and are subject to strict privacy regulations that prohibit public deposition, but are available from the corresponding author on reasonable request.

## Abbreviations

AUC: Area under curve
CA199: Carbohydrate antigen 199
CDFI: Colour Doppler flow imaging
CEA: Carcinoembryonic antigen
Grad-CAM: Gradient-weighted class activation mapping
IDL: Integrative deep learning
IoU: Intersection over union
