# Supplementary material for: An integrative deep learning model based on dual-mode ultrasound for diagnosing gallbladder polyps
Source: Insights Imaging. 2026 Feb 2;17:32. doi: 10.1186/s13244-026-02213-8 (PMC12864583; doi:10.1186/s13244-026-02213-8)
Supplement: Supplementary file 1 — ELECTRONIC SUPPLEMENTARY MATERIAL [file 13244_2026_2213_MOESM1_ESM.pdf]

An Integrative Deep Learning Model Based on Dual-mode Ultrasound for Diagnosing Gallbladder Polyps

ELECTRONIC SUPPLEMENTARY MATERIAL

Appendix 1

After fasting for more than eight hours, patients underwent ultrasound examination in the left lateral position. Ultrasound images were obtained using four ultrasound scanners by experienced US radiologists. Four ultrasound scanners were listed below: ARIETTA70 (Hitachi Medical Systems) with a C251 convex transducer (1-5MHz); GE Healthcare LOGIQ E9 (GE healthcare) with a C1-6 convex transducer (1-6MHz); Epiq7 (Philips Medical Systems) with a C5-1 convex transducer (1-5MHz); Resona 7s (Mindray Medical Systems) with a SC5-1U convex transducer (1-5MHz). If a patient had multiple lesions, the largest poly was selected for observation.

For grey-scale ultrasound, ultrasound radiologists scanned the gallbladder area of patients to obtain grey-scale ultrasound images of multiple slices of the gallbladder polyps, and recorded the images with the largest diameter of the polyps. For CDFI, the colour scale was set as -10-10cm/s. Ultrasound radiologists selected the appropriate slices in CDFI mode, and records the blood flow inside and around the polyps by adjusting the colour gain and sampling frame.

Appendix 2

(The experimental code has been publicly available on GitHub. For access, please refer to the link: https://github.com/daisy0511/An-Integrative-Deep-Learning-Model-Based-on-Dual-mode-Ultrasound-for-Diagnosing-Gallbladder-Polyps.git)

Initially, we applied uniform preprocessing operations to all images, including resizing to a consistent dimension and normalization. For grey-scale ultrasound images, homomorphic filtering was first applied to attenuate speckle noise, followed by histogram equalization to enhance image contrast, thereby improving subsequent segmentation and classification outcomes. Similarly, for colour Doppler ultrasound images, homomorphic filtering was employed to reduce speckle noise, and histogram equalization was used to boost contrast. Moreover, to further augment the dataset, various data augmentation techniques were utilized, including horizontal and vertical flipping, random rotation, and translation. These techniques increased the diversity of the training data and the generalization ability of the model.

Appendix 3

First, we obtain segmentation masks of the gallbladder from the inference results produced by a trained segmentation model. Based on these segmentation masks, we generate bounding boxes, which are the smallest rectangles enclosing the target regions. To ensure more comprehensive information coverage, we expand these bounding boxes by 0.15 times the length of their longest side. This step effectively determines the location and extent of the gallbladder region while ensuring that critical surrounding information is not omitted. Once the bounding boxes are generated and expanded, we crop the grey-scale and blood flow images according to these bounding boxes, extracting the target regions from the original images. The extracted regions serve as the ROIs and typically contain areas of greatest interest to clinicians or researchers, such as tumors, pathological tissues, or blood flow conditions.

Appendix 4

The extracted feature vectors h_{M_1} and h_{M_2} from ResNeXt34 and ResNet101, respectively, are then fed into the Fusion-Block for feature fusion. Within the fusion module, the extracted features h_{M_1} and h_{M_2} undergo normalization to ensure they are on the same scale for seamless fusion. Subsequently, a linear transformation is applied to map the input feature vectors to the $Query(Q)$, $Key(K), Value(V)$ spaces.

$$Q_{M_1} = W_Q^{M_1} \cdot h_{M_1},\ K_{M_1} = W_K^{M_1} \cdot h_{M_1},\ V_{M_1} = W_V^{M_1} \cdot h_{M_1}$$

$$Q_{M_2} = W_Q^{M_2} \cdot h_{M_2},\ K_{M_2} = W_K^{M_2} \cdot h_{M_2},\ V_{M_2} = W_V^{M_2} \cdot h_{M_2}$$

where, W_Q, W_K and W_V are the weight matrices for the query, key, and value, respectively. Next, the dot product of the query and key vectors is computed and normalized through the softmax function to obtain the attention weights.

$$Attention(Q, K, V) = softmax\left(\frac{QK^T}{\sqrt{d_k}}\right)V$$

Specifically, we calculate the self-attention weights for the features of grey-scale ultrasound images and colour Doppler ultrasound images separately.

$$A_{M_1} = softmax\left(\frac{Q_{M_1}K_{M_1}^T}{\sqrt{d_k}}\right),\ A_{M_2} = softmax\left(\frac{Q_{M_2}K_{M_2}^T}{\sqrt{d_k}}\right)$$

To achieve cross-modal information fusion, we also need to calculate the attention weights between the grey-scale ultrasound and CDFI.

$$A_{M_1 \to M_2} = softmax\left(\frac{Q_{M_1}K_{M_2}^T}{\sqrt{d_k}}\right),\ A_{M_2 \to M_1} = softmax\left(\frac{Q_{M_2}K_{M_1}^T}{\sqrt{d_k}}\right)$$

The attention weights are then used to perform a weighted sum operation on the value vectors, resulting in the fused feature vectors:

$$h_{F_1} = A_{M_1}V_{M_1} + A_{M_1\to M_2}V_{M_2}$$
$$h_{F_2} = A_{M_2}V_{M_2} + A_{M_2\to M_1}V_{M_1}$$

Finally, the two fused feature vectors are concatenated to form the final fused feature vector h_F:

$$h_F = [h_{F_1}; h_{F_2}]$$

Then, the fused feature vector h_F is fed into our designed classification head.

The classification head is responsible for the benign-malignant classification of the fused features. It consists of several simple linear layers (fully connected layers) to maintain a straightforward and efficient model structure. The input to the classification head is the fused feature vector h_F, which contains comprehensive information from both grey-scale ultrasound and CDFI. First, the fused feature vector is fed into the first fully connected layer, typically set with an output dimension of 512, utilizing the ReLU activation function, with an optional Dropout layer to prevent overfitting. Next, the features pass through the second and third fully connected layers, with gradually decreasing output dimensions, also using ReLU activation and optional Dropout layers. Finally, the processed features are input to the output layer, which has an output dimension of 2, employing the Softmax activation function to convert the outputs into a probability distribution.

Appendix 5

First, feature maps from a convolutional layer are extracted, and gradients of the target class prediction score with respect to these feature maps are computed. Subsequently, global average pooling is applied to the gradients across spatial dimensions, yielding channel-wise weights that quantify each channel's contribution to the target class. These weights are then multiplied with their corresponding feature map channels and summed to generate the raw class activation map (CAM). Following ReLU activation to retain only positive relevance, the activated map is upsampled to the input image dimensions. Finally, pseudo-colour mapping is applied to produce the resultant heatmap.

Supplementary Tables

Table S1 Univariate analysis and multivariate regression analysis for neoplastic polyps

Characteristics	non-neoplastic polyps (n=117)	neoplastic polyps (n=113)	Univariate analysis	Multivariate analysis		
			p value	OR value	95%CI	*p* value
**Clinical characteristics**						
Sex			0.332			
Female	63	68				
Male	54	45				
Age(y)	47.10±14.56	60.37±14.30	<0.01	1.03	1.00~1.06	0.02
CA199(U/ml)			<0.01			0.26
CEA(ng/ml)			<0.01			0.99
Gastrointestinal symptom			<0.01			0.24
Present	27	54				
Absent	90	59				
**US characteristics**						
Size of polyps(mm)	10.16±3.68	21.35±8.94	<0.01	1.31	1.18~1.45	<0.01
Number			<0.01			0.09
Single	57	87				
Multiple	60	26				
Colour Doppler signal			<0.01			0.43
Present	50	92				
Absent	67	21				
Echogenicity			0.42			
Hypoechoic	33	41				
Isoechoic	63	53				
Hyperechoic	21	19				
Gallbladder Stone			<0.01	3.50	1.16~10.59	0.03
Present	13	42				
Absent	104	71				
Stalk of polyps			<0.01	4.94	2.15~11.38	<0.01
Thin stalk or ball on the wall	83	13				
Wide/thick stalk or sessile	34	100				
Focal wall thickening			<0.01			0.05
Present	1	45				
Absent	116	68				

CA199= Carbohydrate antigen 199; CEA= Carcinoembryonic antigen

Table S2 Univariate analysis and multivariate regression analysis for malignant polyps

Characteristics	benign polyps (n=163)	malignant polyps (n=67)	Univariate analysis	Multivariate analysis		
			p value	OR value	95%CI	*p* value
**Clinical characteristics**						
Sex			0.41			
Female	90	41				
Male	73	26				
Age(y)	49.07±14.76	64.70±12.73	<0.01	1.03	1.00~1.07	0.045
CA199(U/ml)			<0.01			0.11
CEA(ng/ml)			<0.01			0.37
Gastrointestinal symptom			<0.01			
Present	44	37				0.85
Absent	119	30				
**US characteristics**						
Size of polyps(mm)	11.69±5.33	25.31±8.09	<0.01	1.20	1.11~1.30	<0.01
Number			0.01			
Single	91	53				0.83
Multiple	72	14				
Colour Doppler signal			<0.01			
Present	84	58				0.09
Absent	79	9				
Echogenicity			<0.01			0.13
Hypoechoic	41	33				
Isoechoic	88	28				
Hyperechoic	34	6				
Gallbladder Stone			<0.01			0.91
Present	26	29				
Absent	137	38				
Stalk of polyps			<0.01	5.64	1.17~27.26	0.03
Thin stalk or ball on the wall	94	2				
Wide/thick stalk or sessile	69	65				
Focal wall thickening			<0.01	4.38	1.42~13.52	0.01
Present	7	39				
Absent	156	28				

CA199= Carbohydrate antigen 199; CEA= Carcinoembryonic antigen

Table S3 The unnecessary cholecystectomy rate of each model

	Sensitivity [a]	Misdiagnosed rate of neoplastic polyp	Unnecessary cholecystectomy rate
Validation set			
IDL model	85.0%	22.7%(5/22)	15.0%
SRU guidelines	(17/20)	9.1%(2/22)	28.6%
Size≥10mm	71.4%	4.5%(1/22)	38.2%
	(20/28)		
	61.8%		
	(21/34)		
Test set			
IDL model	78.6%	21.4%(6/28)	21.4%
SRU guidelines	(22/28)	7.1%(2/28)	40.9%
Size≥10mm	59.1%	0%(0/28)	45.1%
	(26/44)		
	54.9%		
	(28/51)		

IDL= Integrative deep learning; SRU = Society of Radiologists in Ultrasound

[a] Percentage of patients with model-diagnosed neoplastic polyps requiring surgical intervention

Supplementary Figure1

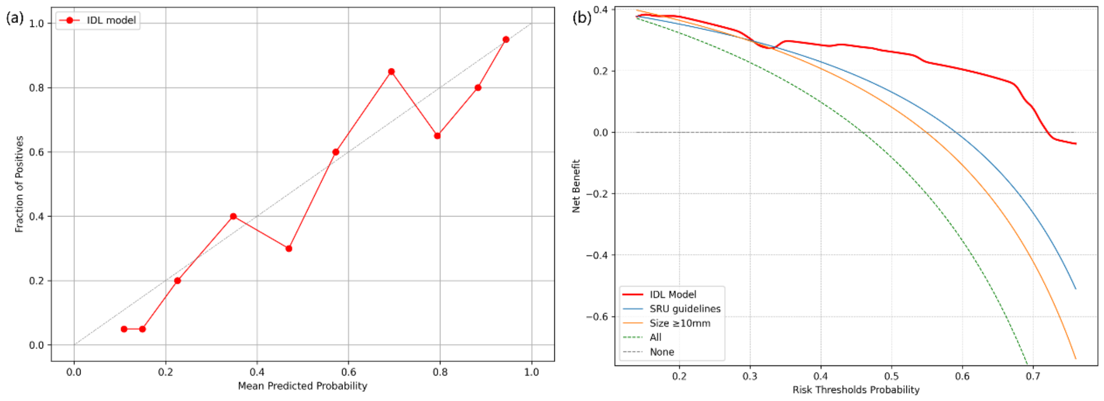

FigureS1. Diagnostic performance of integrative deep learning (IDL) model. (a) The calibration curves of the IDL model for neoplastic polyps (polyps that suggested surgical consultation) in validation set. (b)The decision curve analysis of three models in test set. Note: For the two binary models lacking inherent probability outputs, we defined the predicted negative results of the Society of Radiologists in Ultrasound (SRU) guidelines and size ≥10mm as a probability of 0.1, and the predicted positive results as 0.9 for calculation.
